# Supplementary material for: Benzaldehyde Attenuates the Fifth Stage Larval Excretory–Secretory Product of Angiostrongylus cantonensis-Induced Injury in Mouse Astrocytes via Regulation of Endoplasmic Reticulum Stress and Oxidative Stress
Source: Biomolecules. 2022 Jan 21;12(2):177. doi: 10.3390/biom12020177 (PMC8961544; doi:10.3390/biom12020177)
Supplement: Supplementary file 1 [file biomolecules-12-00177-s001.zip › Supplementary File(s)/Figure S1.pdf]

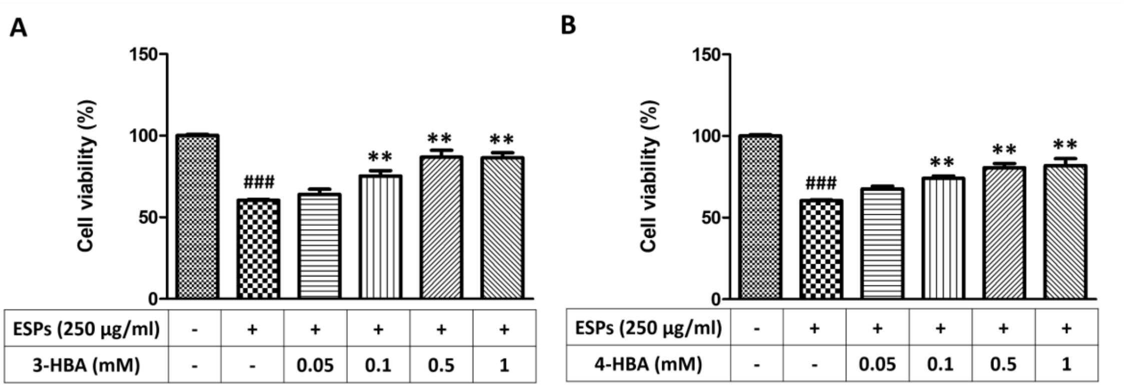

Figure S1. The effects of 3-HBA and 4-HBA in astrocytes treated with *A. cantonensis* L5 ESPs. Cells were pretreated with different concentration of (A) 3-HBA and (B) 4-HBA (0.05, 0.1, 0.5 and 1 mM) and then treated with A. cantonensis L5 ESPs (250 µg/ml). Cell viability was subsequently measured by the CCK8 assay (n=3). $^{\#\#\#}P <$ 0.001, compared to control. *P< 0.05, **P< 0.01, compared to cells exposed to ESPs.
